# Books and Pamphlets Received

**Published:** 1886-10-20

**Authors:** 


### BOOKS AND PAMPHLETS RECEIVED.

*Clinical Note, on Uterine Surgery.* By J. Marion Sims, A. B., M. D., late Surgeon to the Woman's Hospital, New York. New York : William Wood & Company.

The practitioner who has not read Sims knows little of Gynæcology, its history, developments, wonderful achievements, and undisputable claims to the careful attention and study of a progressive medical mind. To ignore Sims is to ignore a science which is as self-asserting as that of Anatomy.

We are in receipt of the above named work. We have read it, not only with delight but with profit, and trust our readers, old and young, adepts or tyros, will secure a copy. We promise them a rare treat, much interest, and most valuable information. Price, only $1.

